# P-1955. Risk Factors Associated with Increased Mortality in Candida parapsilosis Candidemia (RAIMCp)

**DOI:** 10.1093/ofid/ofaf695.2123

**Published:** 2026-01-11

**Authors:** Marlene Garcia, Felix Bratosin, Adam Caulfield, Kevin Fitzgerald, James Lim, Gordana Simeunovic

**Affiliations:** Corewell Health /Michigan State University, Grand Rapids, MI; Corewell Health West/Michigan State University, Grand Rapids, Michigan; Corewell Health, Grand Rapids, Michigan; Corewell Health, Grand Rapids, Michigan; Corewell health, Grand Rapids, Michigan; Corewell Health/ Michigan State University, grand rapids, Michigan

## Abstract

**Background:**

*Candida parapsilosis (CP)* is one of the most common *Candida* species isolated from bloodstream infections (15-30%) with mortality rates reaching up to 36%. Scarce data exists on risk factors associated with CP candidemia mortality. In this retrospective study we evaluate patient population and outcomes associated with CP candidemia to identify risk factors for severe disease.

**Figure 1.**
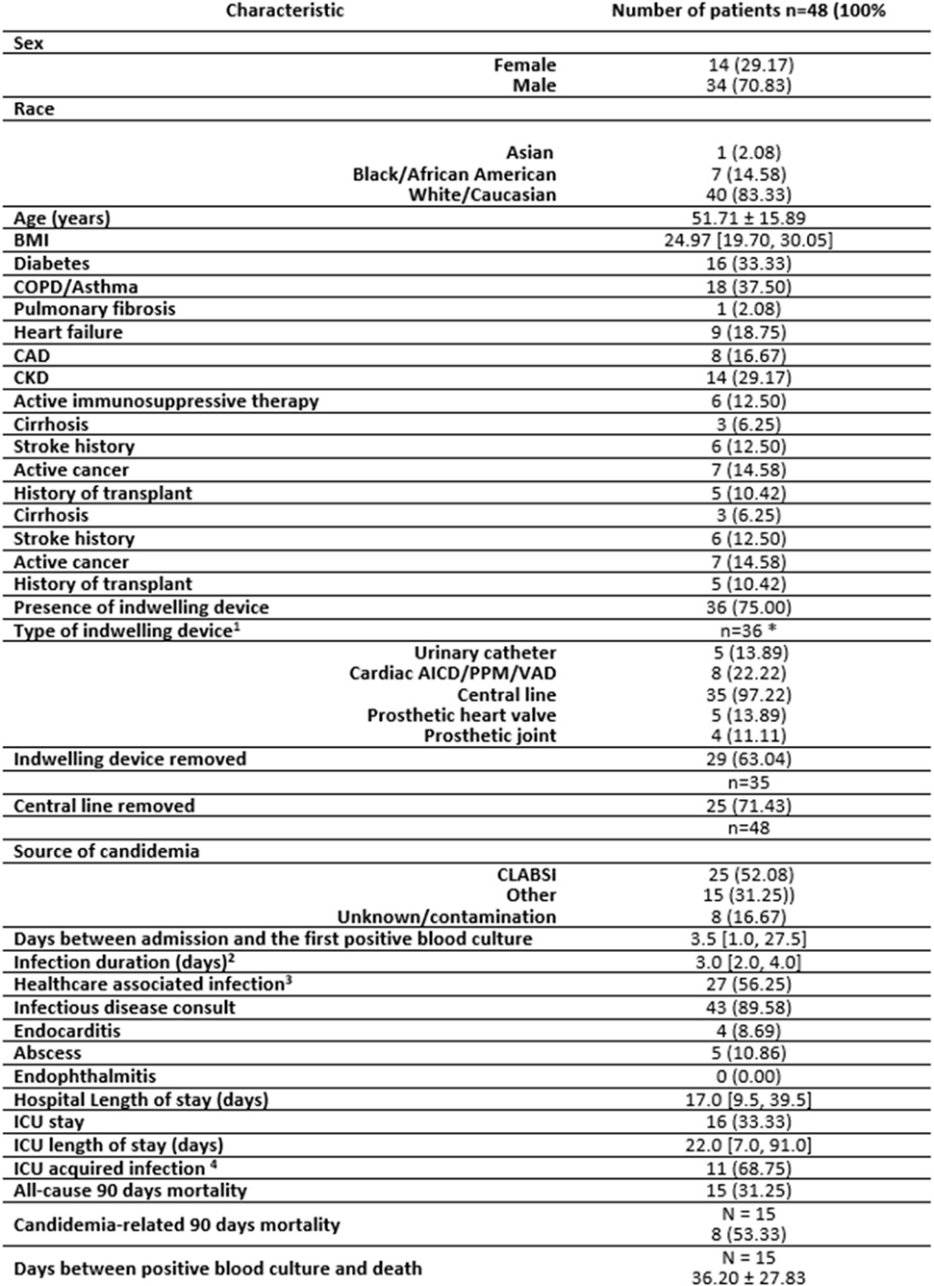
Patient demographics, comorbidities, clinical courses and outcomes. 1Patients can have more than one device; 2Infection duration is defined as the number of days between the first positive and first negative culture; 3 healthcare associated infection is defined by blood cultures being positive more than 48hrs after admission to the hospital; 4 ICU acquired infection is defined by blood culture being positive more than 48hrs after admission to the ICU.

**Figure 2.**
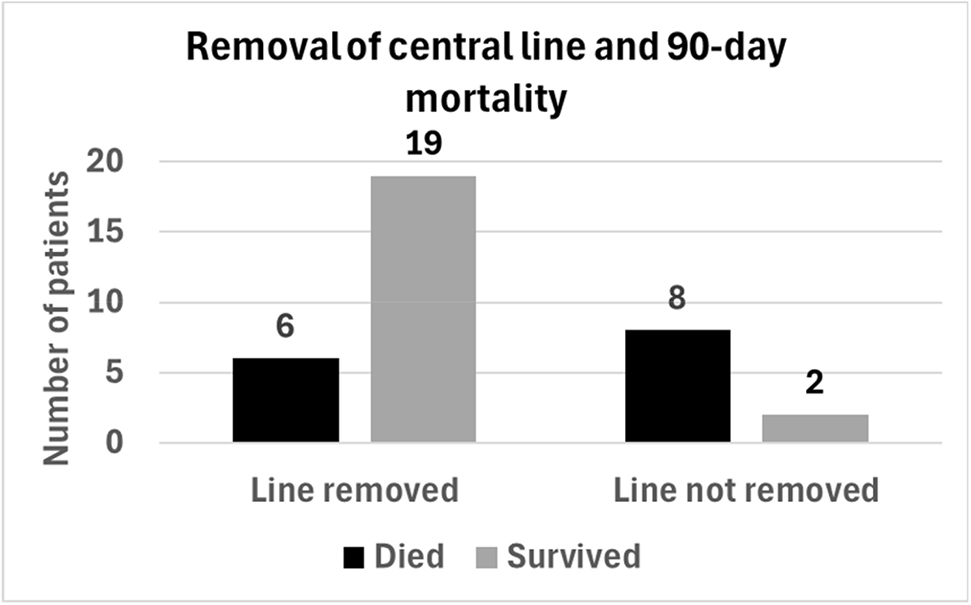
Central line removal and 90-day mortality. 35/48 (72.92%) patients with candidemia had a central line in place. 25 patients had central line removed, and 19/25 (76%) survived. 8/10 patients did not have central line removed, and only 2/10 (20%) survived (p= 0.00225).

**Methods:**

This is a retrospective chart review of adult patients hospitalized at Corewell Health West in Michigan with at least one positive blood culture for *Candida parapsilosis* between November 1^st^, 2017 and January 29^th^, 2025. We identified patients’ demographics, comorbidities and clinical course. Evaluated outcomes included hospital and intensive care unit (ICU) length of stay (LOS), and 90 days all-cause mortality. We examined patients’ characteristics according to mortality status using Chi-squared and Fisher exact tests for categorical and Wilcoxon Rank Sum test for numeric variables to assess statistical significance using an alpha of p< 0.05.

**Figure 3.**
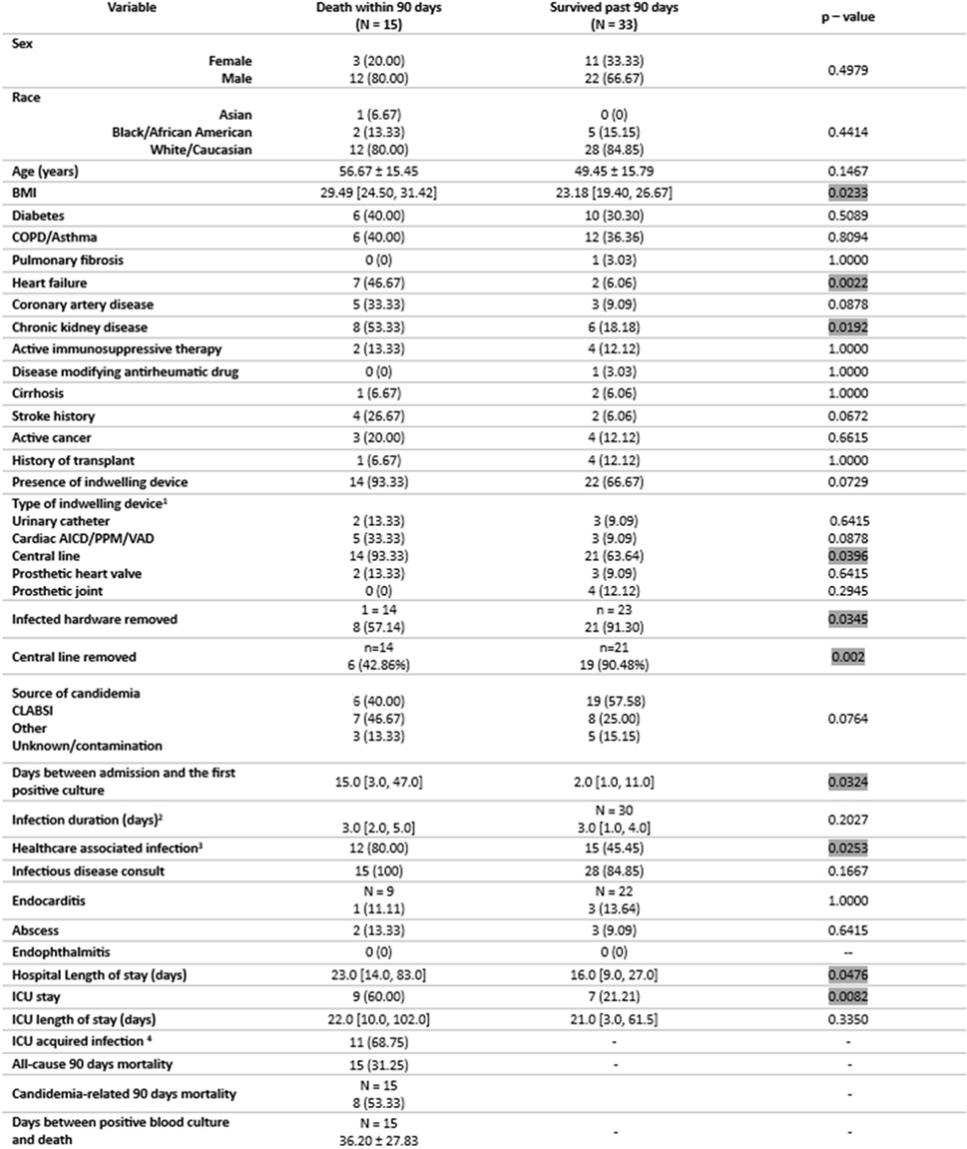
Characteristics and outcomes by 90-day mortality status. 1Patients can have more than one device; 2Infection duration is defined as number of days between the first positive and first negative culture; 3 healthcare associated infection is defined by blood cultures being positive more than 48hrs after admission to the hospital; 4 ICU acquired infection is defined by blood culture being positive more than 48hrs after admission to the ICU.

**Figure 4.**
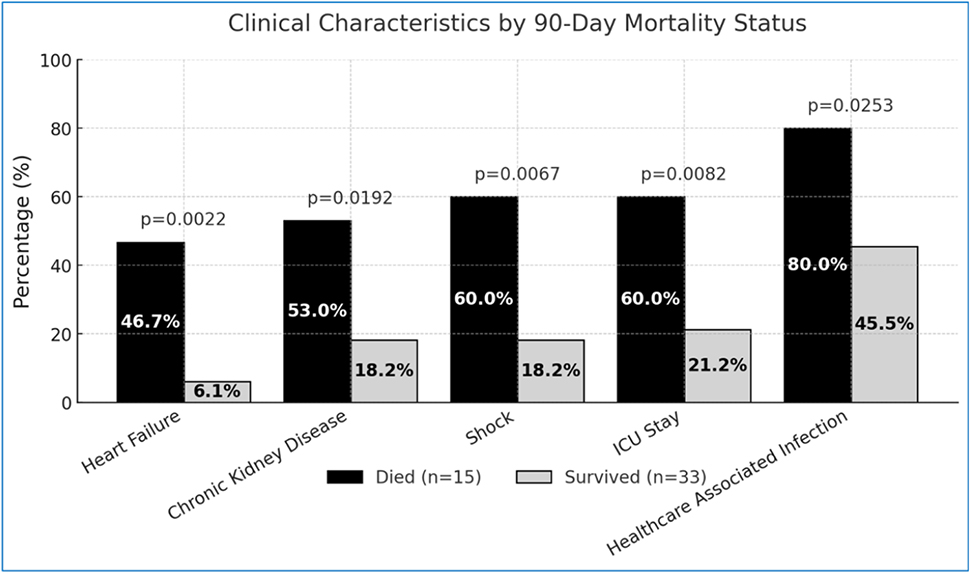
Characteristics significantly associated with mortality.

**Results:**

15/48 (31.25%) patients died within 90 days, 8 of which had a cause of death attributed directly to candidemia. 35/48 (72.92%) patients had a central line (CL) in place. CL was determined to be the source of CP candidemia in 25/48 (52.08%) patients (Figure 1). In patients with CL, removal had a significant positive impact on survival (p= 0.002) (Figure 2). Deceased patients were more likely to have obesity (p= 0.02), heart failure (p= 0.002), chronic kidney disease (p= 0.02), CL in place (p= 0.04), healthcare associated infection (p= 0.02), shock (p=0.006), ICU stay (p= 0.008) (Figure 3 and Figure 4).

**Conclusion:**

Our data identifies several risk factors that may affect mortality in patients with CP candidemia, particularly pointing out the relationship between the presence of CL and CP candidemia and lower mortality associated with CL removal.

**Disclosures:**

All Authors: No reported disclosures

